# Effects of Indoleamine 2,3-Dioxygenase Deficiency on High-Fat Diet-Induced Hepatic Inflammation

**DOI:** 10.1371/journal.pone.0073404

**Published:** 2013-09-09

**Authors:** Junji Nagano, Masahito Shimizu, Takeshi Hara, Yohei Shirakami, Takahiro Kochi, Nobuhiko Nakamura, Hirofumi Ohtaki, Hiroyasu Ito, Takuji Tanaka, Hisashi Tsurumi, Kuniaki Saito, Mitsuru Seishima, Hisataka Moriwaki

**Affiliations:** 1 Department of Internal Medicine, Gifu University Graduate School of Medicine, Gifu, Japan; 2 Department of Informative Clinical Medicine, Gifu University Graduate School of Medicine, Gifu, Japan; 3 Department of Tumor Pathology, Gifu University Graduate School of Medicine, Gifu, Japan; 4 Human Health Sciences, Graduate School of Medicine and Faculty of Medicine, Kyoto University, Kyoto, Japan; Centro de Investigación en Medicina Aplicada (CIMA), Spain

## Abstract

Hepatic immune regulation is associated with the progression from simple steatosis to non-alcoholic steatohepatitis, a severe condition of inflamed fatty liver. Indoleamine 2,3-dioxygenase (IDO), an intracellular enzyme that mediates the catabolism of L-tryptophan to L-kynurenine, plays an important role in hepatic immune regulation. In the present study, we examined the effects of IDO gene silencing on high-fat diet (HFD)-induced liver inflammation and fibrosis in mice. After being fed a HFD for 26 weeks, the IDO-knockout (KO) mice showed a marked infiltration of inflammatory cells, especially macrophages and T lymphocytes, in the liver. The expression levels of F4/80, IFNγ, IL-1β, and IL-6 mRNA in the liver and the expression levels of F4/80 and TNF-α mRNA in the white adipose tissue were significantly increased in IDO-KO mice, although hepatic steatosis, the accumulation of intrahepatic triglycerides, and the amount of oxidative stress were lower than those in IDO-wild-type mice. IDO-KO mice also developed marked pericellular fibrosis in the liver, accumulated hepatic hydroxyproline, and exhibited increased expression levels of hepatic TGF-β1 mRNA. These findings suggest that IDO-KO renders the mice more susceptible to HFD-induced hepatic inflammation and fibrosis. Therefore, IDO may have a protective effect against hepatic fibrosis, at least in this HFD-induced liver injury model.

## Introduction

Non-alcoholic fatty liver disease (NAFLD), which is strongly associated with obesity and metabolic syndrome, is one of the most common causes of chronic liver disease worldwide. NAFLD includes a spectrum of disturbances that encompasses various degrees of liver damage ranging from non-alcoholic steatohepatitis (NASH), a severe condition of inflamed fatty liver that can progress to hepatic fibrosis, cirrhosis, or even hepatocellular carcinoma. The critical features of NASH, in addition to simple steatosis, include forms of hepatocellular degeneration such as ballooning and Mallory hyaline degeneration, mixed inflammatory cell infiltration, and the development of fibrosis [1,2]. Obesity is associated with chronic low-grade systemic inflammation, which contributes to the progression from hepatic steatosis to NASH [3]. Among various immune cells, T lymphocytes play a critical role in the induction of inflammation both in the liver and in white adipose tissue (WAT) [4,5]. Macrophage infiltration into WAT is also an early contributing event in the development of systemic inflammation because it is accompanied by tumor necrosis factor (TNF)-α production, a central mediator of the inflammatory response [6]. These reports, therefore, indicate that chronic inflammation plays a key role in the pathogenesis of NASH [7].

Indoleamine 2,3-dioxygenase (IDO), an intracellular enzyme that degrades the essential amino acid L-tryptophan along the L-kynurenine pathway, is induced during inflammation by several immune factors, including interferon (IFN) γ [8]. IDO is considered to exert powerful immunomodulatory effects, including the promotion of immune tolerance, because L-kynurenine and some other metabolites derived from tryptophan by IDO can inhibit T cell activation and proliferation while increasing immunosuppressive regulatory T-cell (Tregs) activity [9–11]. The liver is a special lymphoid organ and is thus particularly susceptible to injury as a result of the immune response, which is primarily mediated by T lymphocytes [12]. IDO is activated in infectious, autoimmune, and malignant diseases that involve cellular immune activation in various organs, including the liver [13]. In fact, upregulation of the IDO expression in the liver and increased serum IDO activity have been found in chronic hepatitis C patients [14,15]. The IDO expression is also enhanced in the liver and adipose tissue in obese individuals [16].

Several rodent studies have revealed the role of IDO in liver injury. In hepatitis B virus (HBV) transgenic mice, the IDO expression in hepatocytes is enhanced in mice with liver injury caused by HBV-specific cytotoxic T lymphocytes [17]. Inhibition of IDO activity exacerbates liver injury in both α-galactosylceramide- and carbon tetrachloride (CCl_4_)-induced acute hepatitis models and is associated with the induction of TNF-α [18,19]. These reports suggest that IDO plays a critical role in the regulation of liver inflammation and that targeting IDO activity might be an effective strategy for attenuating acute liver injury. However, the role of IDO in steatosis-induced liver injury has not yet been clarified. In the present study, we examined the effects of IDO on high-fat diet (HFD)-induced liver steatosis and subsequent hepatic inflammation and fibrosis using IDO-deficient mice.

## Materials and Methods

### 2.1 Animals and experimental procedure

This study was carried out in strict accordance with the recommendations of the Guide for the Care and Use of Laboratory Animals of Gifu University Life Science Research Center. The protocol was approved by the Committee on the Ethics of Animal Experiments of Gifu University (Permit Number: 24-65). All surgeries were performed under sodium pentobarbital anesthesia, and all efforts were made to minimize animal suffering. Five-week-old male IDO-wild-type (WT) mice and IDO-knockout (KO) mice with a C57BL/6J background were obtained from The Jackson Laboratory (Bar Harbor, ME, USA). HFD-60 (506.2 kcal/100 g) with 62.2% of the calories derived from fat was purchased from Oriental Yeast (Tokyo, Japan). The cholesterol content of the diet was 33.0 mg/100 g. After 1 week of acclimatization, 8 WT mice and 8 KO mice were given a pelleted HFD throughout the experiment (26 weeks) with free access to tap water and food. At the end of the experiment (32 weeks of age), all mice were sacrificed under sodium pentobarbital anesthesia and the livers were carefully removed.

### 2.2 Histopathological and immunohistochemical examinations

For all the experimental mice, 4 μm-thick sections of formalin-fixed and paraffin-embedded livers were stained with hematoxylin & eosin (H&E) for conventional histopathology or with Sirius Red stain to determine the presence of liver fibrosis. The histological features of the livers were evaluated using the NAFLD activity score (NAS) system [20]. The computer-assisted quantitative analyses of hepatic fibrosis development were carried out using the BZ-Analyzer-II software program (KEYENCE, Osaka, Japan) [21,22].

In order to evaluate the infiltration of inflammatory cells in the liver, immunohistochemical staining for Mac-1 (a macrophage marker), CD3 (a T-cell marker), and NIMP-R14 (a neutrophil marker) of paraffin-embedded sections was performed using the linked streptavidin-biotin method. Rat monoclonal anti-Mac-1 antibody (MAB1387Z, 1:50 dilution) was purchased from Chemicon Iuternational (Temecula, CA, USA). Rabbit polyclonal anti-CD3 (ab5690, 1:100 dilution) antibodies and rat monoclonal anti-neutrophil antibody (NIMP-R14, ab2557, 1:50 dilution) were obtained from Abcam (Cambridge, MA, USA). On the Mac-1-, CD3-, and NIMP-R14-immunostained sections, the inflammatory cells that intensively reacted to these antibodies were counted and the data are expressed as the percentage of total inflammatory cells in the liver. A positive cell index (%) was determined by counting at least 500 cells in a section from each mouse.

### 2.3 Hepatic hydroxyproline analysis

The hepatic hydroxyproline content (μmol/g wet liver) was quantified colorimetrically in duplicate samples from approximately 200mg of the wet-weight liver tissues, as described previously [22].

### 2.4 RNA extraction and quantitative real-time RT-PCR analysis

Total RNA was isolated from the livers and adipose tissue of the mice using the RNeasy Mini Kit and RNeasy Lipid Tissue Mini Kit (Qiagen, Hilden, Germany), respectively [21]. cDNA was amplified from 0.5 μg of total RNA using the SuperScript III First-Strand Synthesis System (Invitrogen, Carlsbad, CA, USA). A quantitative real-time reverse transcription-PCR (RT-PCR) analysis was performed using specific primers that amplify F4/80, IFNγ, interleukin (IL)-1β, IL-6, TNF-α, superoxide dismutase (SOD)-1, SOD-2, glutathione peroxidase (GPx), transforming growth factor (TGF)-β1, glyceraldehyde-3-phosphate dehydrogenase (GAPDH), and the ribosomal protein large P0 (RPLP0) genes. The sequences of the primers for these genes, which were obtained from Universal ProbeLibrary Assay Design Center (Roche, Indianapolis, IN, USA), are shown in Table 1. The analysis to quantify the expression levels of tryptophan 2,3-dioxygenase (TDO) was performed using TaqMan Gene Expression Assays (Applied Biosystems, Foster City, CA, USA) and TOYOBO Real-time PCR Master Mix (TOYOBO, Osaka, Japan), as described previously [23]. Each sample was analyzed on a LightCycler Nano (Roche) with FastStart Essential DNA Green Master (Roche). The parallel amplification of GAPDH and RPLP0 was used as the internal control for liver and adipose tissue, respectively.

**Table 1:** Primer sequences.

Gene	Primer sequence	
*SOD1*	F	5’- CAGGACCTCATTTTAATCCTCAC-3’
	R	5’- TGCCCAGGTCTCCAACAT-3’
*SOD2*	F	5’- TGCTCTAATCAGGACCCATTG-3’
	R	5’- GTAGTAAGCGTGCTCCCACAC-3’
*GPx*	F	5’- TTTCCCGTGCAATCAGTTC-3’
	R	5’- TCGGACGTACTTGAGGGAAT-3’
*F4/80*	F	5’- ACAAGACTGACAACCAGACGG-3’
	R	5’- TAGCATCCAGAAGAAGCAGGCGA-3’
*IFNγ*	F	5’- AGCAACAGCAAGGCGAAAAAG-3’
	R	5’- CGCTTCCTGAGGCTGGATTC-3’
*IL-1β*	F	5’- CAAGCAACGACAAAATACCTGTG-3’
	R	5’- AGACAAACCGTTTTTCCATCTTCT-3’
*IL-6*	F	5’- CCGGAGAGGAGACTTCACAGAG-3’
	R	5’- CTGCAAGTGCATCATCGTTGTT-3’
*TNF-α*	F	5’- TGGCCCAGACCCTCACACTCAG-3’
	R	5’- ACCCATCGGCTGGCACCACT-3’
*TGF-β1*	F	5’- ACCGGAGAGCCCTGGATACCA-3’
	R	5’- TATAGGGGCAGGGTCCCAGACA-3’
*RPLP0*	F	5’- ACTGGTCTAGGACCCGAGAAG-3’
	R	5’- CTCCCACCTTGTCTCCAGTC-3’
*GAPDH*	F	5’-GACATCAAGAAGGTGGTGAAGCAG-3’
	R	5’-ATACCAGGAAATGAGCTTGACAAA-3’

### 2.5 Clinical chemistry

The serum levels of alanine aminotransferase (ALT) were measured using a standard clinical automatic analyzer (type 7180; Hitachi, Tokyo, Japan).

### 2.6 Oxidative stress analysis

The serum hydroperoxide levels, one of the markers of oxidative stress, were determined using the derivatives of reactive oxygen metabolites (d-ROM) test (FREE Carpe Diem; Diacron s.r.l., Grosseto, Italy), according to the manufacturer’s protocol.

### 2.7 Determination of the enzymatic activity of IDO

The IDO activity level in the serum was determined by calculating the ratio of the L-kynurenine/L-tryptophan concentrations [23]. Serum samples were deproteinized with 3% perchloric acid. Following centrifugation, aliquots of supernatant were collected to determine the concentrations of L-tryptophan and L-kynurenine using HPLC, as described previously [18].

### 2.8 Hepatic lipid analysis

After total lipids were extracted from the frozen livers (approximately 200 mg), the triglyceride levels were measured using the triglyceride E-test kit (Wako, Osaka, Japan) [21].

### 2.9 Statistical analysis

The data are expressed as the mean ± SD. Statistical significance of the difference between mean values was evaluated using the Mann-Whitney *U* test. Significance was defined as a *P* value less than 0.05.

## Results

### 3.1 General observations

**Figure 1:**
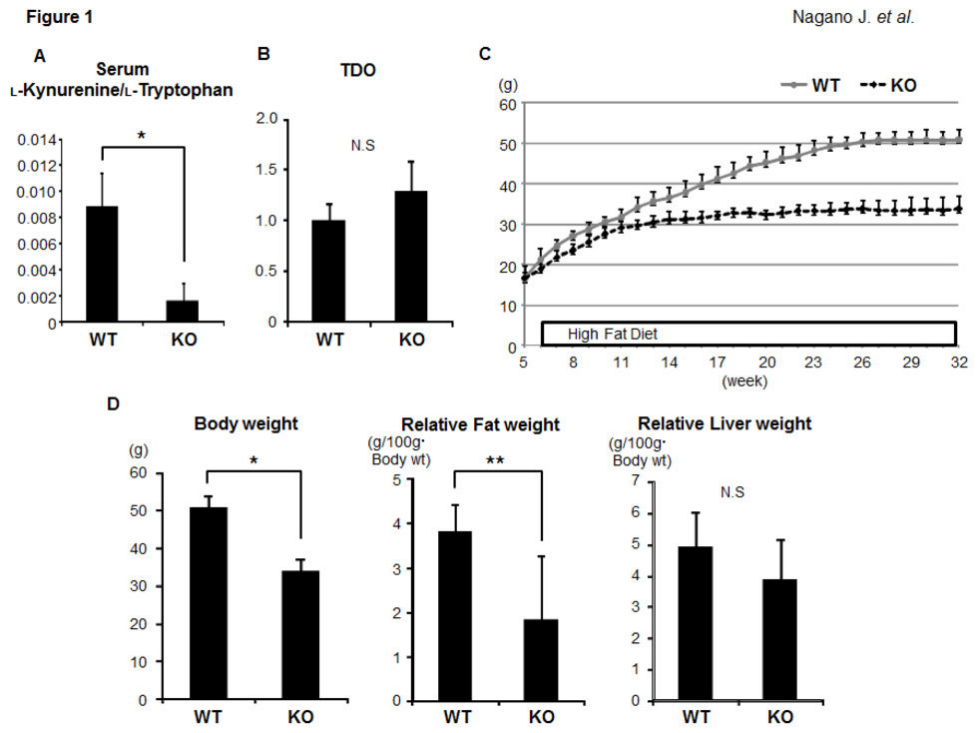
Effects of IDO deficiency on the serum L-kynurenine/L-tryptophan ratio, the expression levels of TDO in the liver, the growth curve, and the body, liver, and fat weights of the experimental mice. (A) The functional IDO activity level was determined by measuring the concentrations of L-kynurenine and L-tryptophan using HPLC. The L-kynurenine/L-tryptophan ratio indicates the IDO activity. (B) Total RNA was isolated from the livers of the experimental mice, and the expression levels of TDO mRNA were examined using quantitative real-time RT-PCR with specific primers. (C) The growth curve of the experimental mice. The body weights of all mice were measured once a week during the experiment. (D) The body weights and relative weights of the adipose tissues and livers of the experimental mice at the termination of the study. The values are expressed as the mean ± SD. * *P* <0.001, ** *P* <0.05.

We initially examined the enzymatic activity of IDO in the serum of the experimental mice by measuring the concentrations of L-kynurenine and L-tryptophan. The L-kynurenine/L-tryptophan ratios in serum of the IDO-KO mice were significantly lower than those in the serum of the IDO-WT mice (Figure 1A, P < 0.001), indicating that IDO activity was clearly inhibited in the IDO-KO mice. TDO, a hepatic enzyme that catalyses the first step of tryptophan degradation, was expressed in the liver in both the IDO-WT mice and the IDO-KO mice; however, IDO deficiency did not have a significant effect on the TDO mRNA expression (Figure 1B). Figure 1C shows the growth curves of the mice during this experiment. The body weight gain of the IDO-KO mice was smaller than that of the IDO-WT mice. At the end of the experiment, the body weights (Figure 1D, P < 0.001) and the relative weights of the adipose tissues of the IDO-KO mice (Figure 1D, P < 0.05) were also significantly lower than those of the IDO-WT mice.

### 3.2 Effects of IDO deficiency on hepatic histopathology in the experimental mice

**Figure 2:**
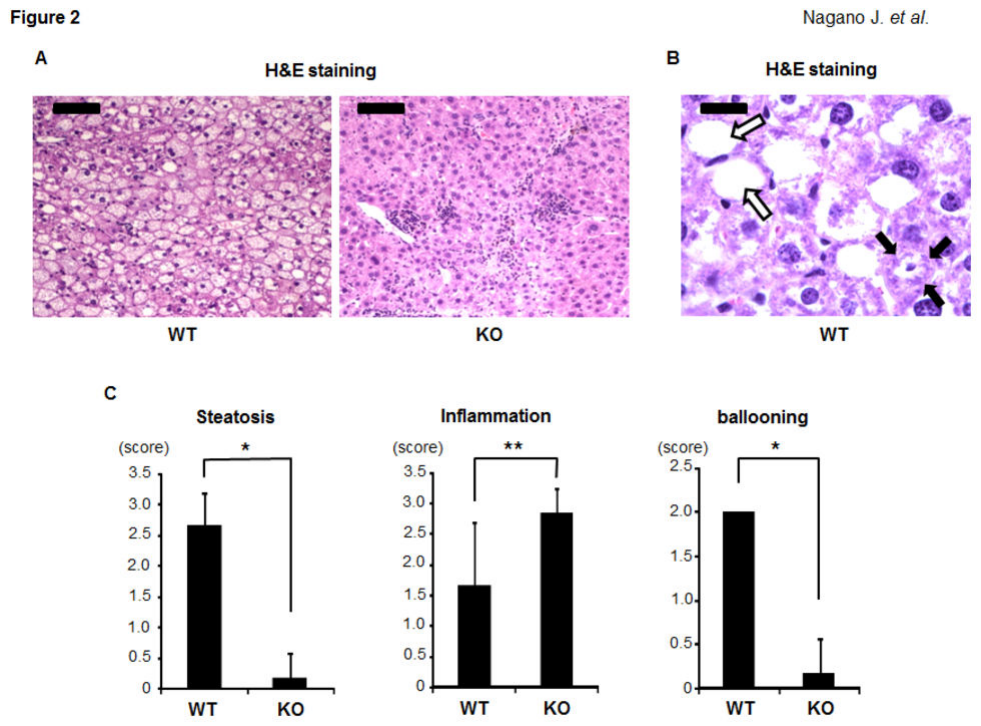
Effects of IDO deficiency on hepatic histopathology in the experimental mice. (A) and (B) H&E staining of liver sections from the experimental mice. (A) Representative photomicrographs of the liver sections of the IDO-WT mice and IDO-KO mice (low-power field). Black bar: 100 μm. (B) An enlarged photo (high-power field) of the liver sections from the IDO-WT mice. Ballooned hepatocytes (indicated by white arrows) and Mallory-Denk bodies (indicated by black arrows) were observed. Black bar: 20 μm. (C) The presence of NAS (steatosis, inflammation, and ballooning) was determined based on the histopathological analysis. The values are expressed as the mean ± SD. * *P* <0.001, ** *P* <0.05.

The H&E staining results of the livers of the IDO-KO mice and IDO-WT mice after 26 weeks of being fed the HFD are presented in Figure 2A and B. The infiltration of inflammatory cells was markedly increased in the livers of the IDO-KO mice, and the NAS inflammation scores were significantly higher than those in the IDO-WT mice (Figure 2C, P < 0.05). Interestingly, the hepatic steatosis and ballooning degeneration of hepatocytes were lower in the IDO-KO mice than in the IDO-WT mice at this experimental time point (Figure 2C, P < 0.001). In addition to the ballooned hepatocytes, Mallory-Denk bodies, which are a recognized feature of alcoholic hepatitis and NASH [24], were also observed in the liver of IDO-WT mice (Figure 2B).

### 3.3 Effects of IDO deficiency on the intrahepatic triglyceride levels, the serum ALT levels, and oxidative stress in the experimental mice

**Figure 3:**
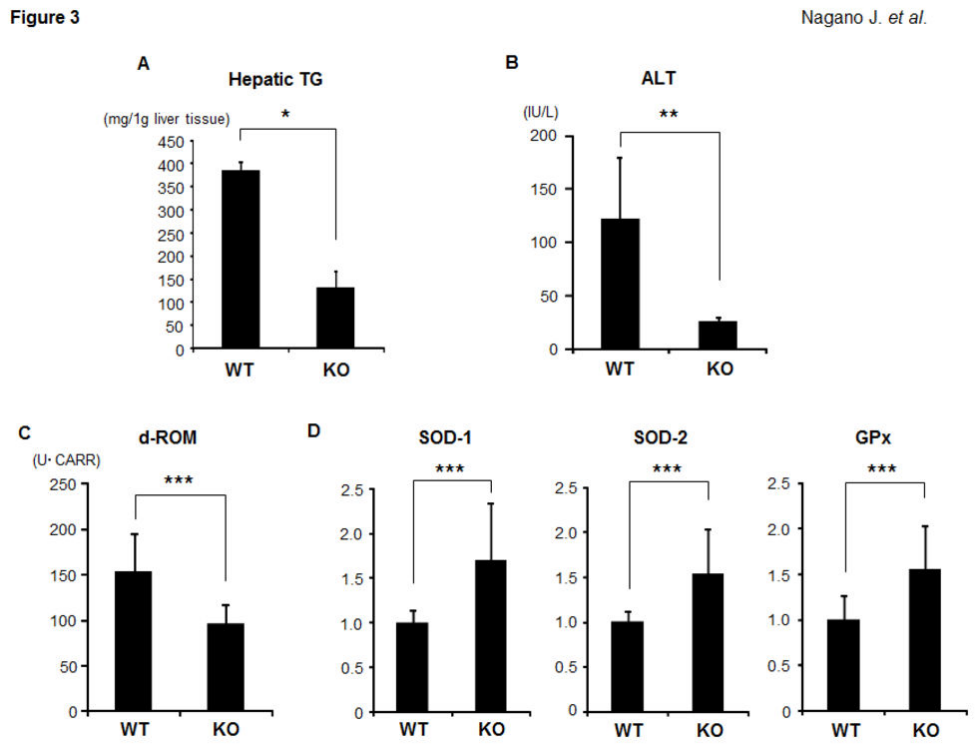
Effects of IDO deficiency on intrahepatic triglycerides, the serum ALT levels, and oxidative stress in the experimental mice. (A) Hepatic lipids were extracted from the frozen livers of the experimental mice, and the triglyceride levels were measured. (B) At sacrifice, blood samples were collected and the serum levels of ALT were assayed. (C) The hydroperoxide levels in the serum at the end of the experiment were determined using the d-ROM test. (D) Total RNA was isolated from the livers of the experimental mice, and the expression levels of SOD-1, SOD-2, and GPx mRNA were examined using quantitative real-time RT-PCR with specific primers. The values are expressed as the mean ± SD. * *P* <0.001, ** *P* <0.01, *** *P* <0.05.

The histological findings were consistent with the measured intrahepatic triglyceride contents: the levels of triglycerides in the livers of the IDO-KO mice were significantly lower than those in the livers of the IDO-WT mice (Figure 3A, P < 0.001). The serum levels of ALT in the IDO-KO mice were also significantly decreased relative to those in the IDO-WT mice (Figure 3B, P < 0.01). In addition, the serum d-ROM levels, which reflect the serum hydroperoxide levels, were significantly lower in the IDO-KO mice than in the IDO-WT mice (Figure 3C, P < 0.05). Compared to the IDO-WT mice, there were also significant increases in the expression levels of SOD-1, SOD-2, and GPx mRNA, which encode antioxidant enzymes, in the livers of the IDO-KO mice (Figure 3D, P < 0.05). These findings indicate that hepatic triglyceride accumulation and oxidative stress are reduced, while antioxidant activity is increased, in mice lacking the IDO gene.

### 3.4 Effects of IDO deficiency on inflammation in the livers and WAT of the experimental mice

**Figure 4:**
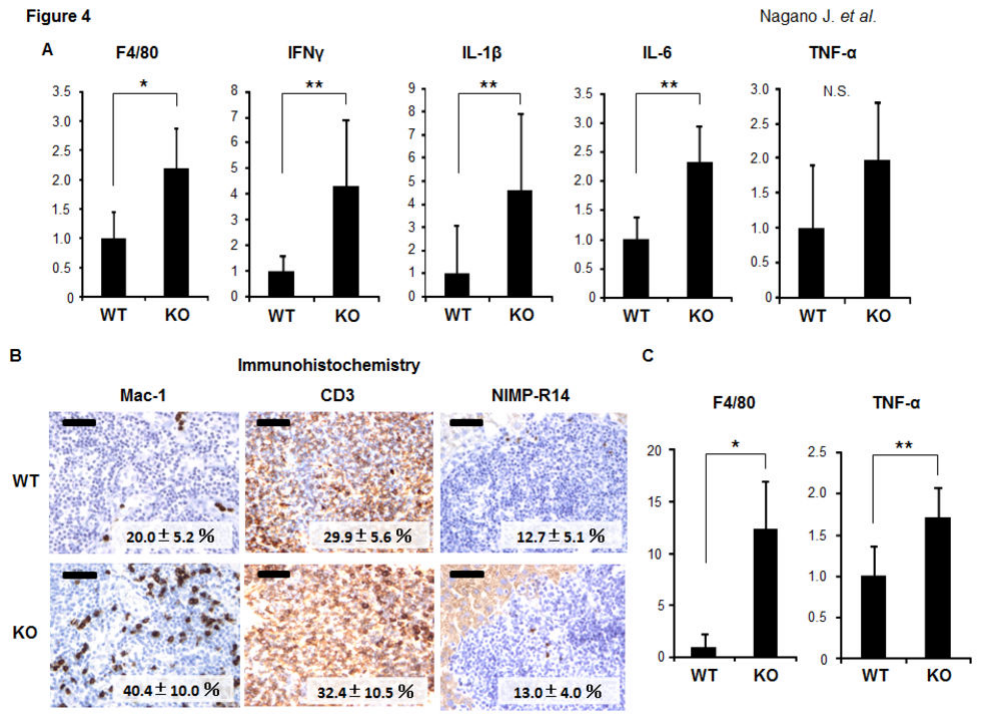
Effects of IDO deficiency on the inflammation in the liver and white adipose tissue of the experimental mice. (A) The expression levels of F4/80, IFNγ, IL-1β, IL-6, and TNF-α mRNA in the livers of the experimental mice. (B) The results of the immunohistochemical analyses of Mac-1, CD3, and NIMP-R14 in the livers of the experimental mice. A positive cell index (%) was shown in each photo. Black bar: 50 μm. (C) The expression levels of F4/80 and TNF-α mRNA in the WAT of the experimental mice. Total RNA was isolated from the livers (A) and WAT (C) of the experimental mice, and the expression levels of each mRNA were examined using quantitative real-time RT-PCR with specific primers. The expression levels of GAPDH mRNA and RPLP0 mRNA were used as internal controls for the liver and WAT, respectively. The values are expressed as the mean ± SD. * *P* <0.01, ** *P* <0.05.

We next examined the expression levels of inflammatory mediators that are implicated in the progression of fatty liver to NASH [7] in the experimental mice. A quantitative real-time RT-PCR analysis revealed that the expression levels of F4/80, a marker of macrophages, were significantly increased in the livers of the IDO-KO mice in comparison to those observed in the livers of the IDO-WT mice (Figure 4A, P < 0.01). There were also significant increases in the expression levels of inflammatory mediators, including IFNγ, IL-1β, and IL-6 mRNA, in the livers of the IDO-KO mice compared to those observed in the livers of the IDO-WT mice (Figure 4A, P < 0.05). The expression levels of TNF-α mRNA were also higher in the livers of the IDO-KO mice than in the livers of the IDO-WT mice; however, the difference was insignificant (Figure 4A). Furthermore, the immunohistochemical analyses demonstrated that the inflammatory cells that had infiltrated into the livers of the IDO-KO mice positively reacted with either the anti-Mac-1(40.4 ± 10.0%) or anti-CD3 (32.4 ± 10.5%) antibodies. On the other hand, the infiltration of neutrophils (13.0 ± 4.0%) was low compared to that of macrophages and T-cells. These findings suggest that macrophages and T lymphocytes were the predominantly increased cell populations in the livers of the IDO-KO mice. The infiltration of Mac-1 positive cells in the livers of IDO-KO mice (40.4 ± 10.0%) was high compared to that of IDO-WT mice (20.0 ± 5.2%) (Figure 4B, P < 0.05), and this is consistent with the results of RT-PCR analysis showing the increased levels of F4/80 mRNA in the livers of IDO-KO mice (Figure 4A).

Moreover, as shown in Figure 4C, the expression levels of F4/80 (*P* < 0.01) and TNF-α(*P* < 0.05) mRNA in WAT were both significantly increased in the IDO-KO mice compared to those observed in the IDO-WT mice, indicating that inflammation is augmented in WAT, in addition to the liver, in the IDO-KO mice [24].

### 3.5 Effects of IDO deficiency on hepatic fibrosis in the experimental mice

**Figure 5:**
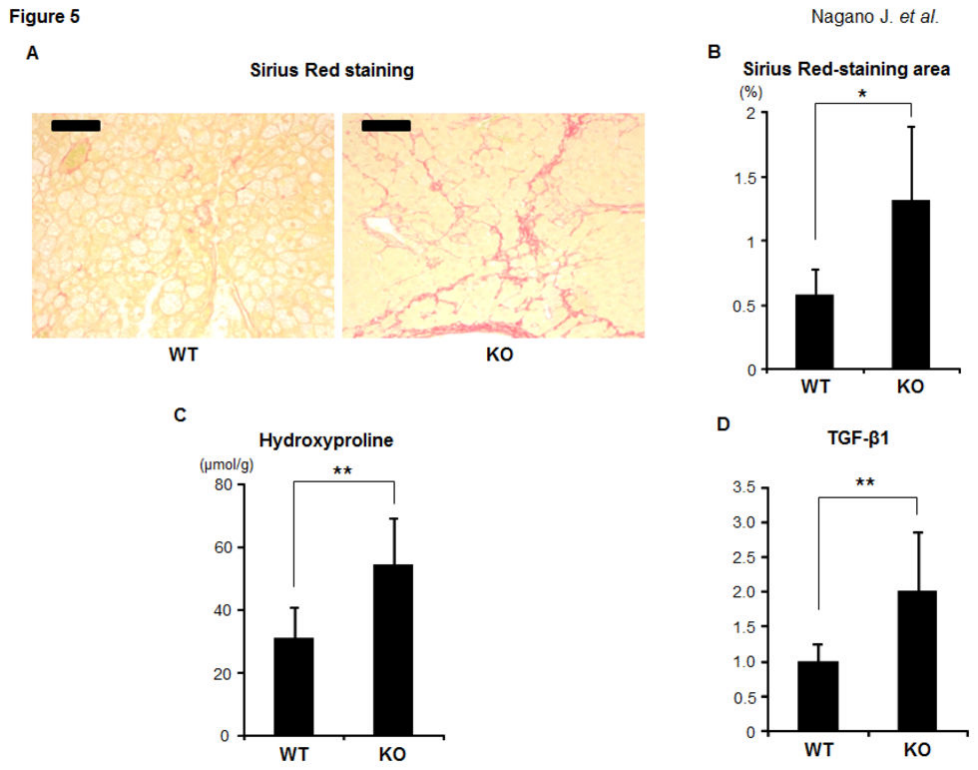
Effects of IDO deficiency on the hepatic fibrosis in the experimental mice. (A) Representative photomicrographs of liver sections stained with Sirius Red to show fibrosis. Black bar: 100 μm. (B) The Sirius Red-stained images of fibrosis were analyzed using a BZ-9000 fluorescence microscope, and the fibrotic area was measured using a BZ-Analyzer-II. (C) The hepatic hydroxyproline contents were quantified colorimetrically. (D) Total RNA was isolated from the livers of the experimental mice, and the expression levels of TGF-β1 mRNA were examined using quantitative real-time RT-PCR with specific primers. The values are expressed as the mean ± SD. * *P* <0.01, ** *P* <0.05.

We next examined whether IDO deficiency has an effect on the development of steatosis-induced hepatic fibrosis. An examination of Sirius Red-stained sections indicated that, compared to the IDO-WT mice, the IDO-KO mice markedly developed pericellular fibrosis in the liver (Figure 5A and B, *P* < 0.01). Similar findings were observed in the measured hepatic hydroxyproline contents: the IDO-KO mice showed a significant increase in the amount of hydroxyproline observed in the liver (Figure 5C, P < 0.05). The expression levels of TGF-β1 mRNA, a central regulator of chronic liver disease contributing to fibrogenesis through inflammation [25], were also remarkably elevated in the livers of the IDO-KO mice compared to those observed in the livers of the IDO-WT mice (Figure 5D, P < 0.05). These findings may indicate that IDO-KO mice are susceptible to the development of steatosis-induced hepatic fibrosis.

## Discussion

The results of the present study indicate that HFD-induced hepatic inflammation and fibrosis are significantly aggravated in IDO-KO mice, although the level of hepatic steatosis and amount of oxidative stress were lower compared to those in IDO-WT mice. Therefore, IDO deficiency is critically involved in the acceleration of hepatic inflammation observed in the present study.

IDO is a rate-limiting enzyme that can degrade tryptophan via the kynurenine pathway. Because the IDO expression and its enzymatic activity, which are tightly controlled by several immune mediators such as IFNγ, play a key role in the suppression of the immune response [8–11], inhibiting the expression and activity of IDO might promote inflammatory signaling. Therefore, based on our present results, we consider that IDO-deficient mice are more susceptible to the induction of inflammation by HFD. Our results are consistent with those of recent reports showing that the inhibition of the enzymatic activity of IDO significantly exacerbated liver injury in α-galactosylceramide (α-GalCer)- and CCl_4_-induced acute hepatitis animal models via the upregulation of IL-6 and TNF-α [18,19]. When the IDO-KO mice were treated with α-GalCer, the production of TNF-α from the infiltrating macrophages in the liver was significantly accelerated, and thus led to the development of severe hepatitis [18]. Therefore, in the present study, the increase in the number of hepatic macrophages might have been critically involved in the exacerbation of HFD-induced hepatic inflammation in the IDO-KO mice. These reports [18,19], together with the results of the present study, suggest that IDO may play a critical role in suppressing excess induction and progression of inflammation in the liver.

Innate immune cells, including Kupffer cells, natural killer T cells, and natural killer cells, play important roles in the excessive production of hepatic T helper 1 cytokines, which is associated with the development of steatohepatitis [4]. The regulation of the immune response by IDO is predominantly based on the ability of IDO to suppress the activation of lymphocytes [9–11]. An increased IDO activity inhibits proliferation and induces apoptosis in T cells and natural killer cells via tryptophan depletion and the production of toxic tryptophan metabolites [9]. In addition, recent studies have revealed that IDO inhibits T cell activation by driving the development of Tregs [10,11]. Tregs, which are actively engaged in the negative control of a variety of immune responses, are recognized as being one of the key players in hepatic immune regulation [26]. HFD-induced steatosis in mice is associated with the depletion of hepatic Tregs and leads to upregulation of the inflammatory pathway [27]. Therefore, an IDO deficiency may increase T cell activation, either directly or indirectly, by suppressing Tregs and thus contributed to a worsening of hepatic inflammation in the present study.

Obesity is associated with systemic low-grade inflammation and immune activation [5,6]. One clinical trial reported that activation of IDO is associated with reduced plasma tryptophan levels in obese patients [28]. IDO is also overexpressed in the liver and adipose tissue in obese subjects [16]. These reports indicate that the overexpression and activation of IDO are implicated in chronic immune activation in obese individuals. T cell infiltration into WAT and subsequent recruitment and activation of macrophages can induce TNF-α production, which is associated with the development of systemic inflammation [5,6]. The present study showed that the expression levels of F4/80 and TNF-α mRNA in WAT are elevated in IDO-KO mice compared to those observed in IDO-WT mice when the mice are fed an HFD, indicating that inflammation of WAT induced by HFD is worsened in IDO deficiency mice. Therefore, our findings suggest that IDO might have the ability to attenuate overactive immune responses caused by obesity in WAT in addition to the liver.

There are some possible limitations associated with the present study. For instance, a recent study demonstrated that neither the overexpression of IDO nor inhibition of its enzymatic activity affected the lipid accumulation in the liver, although the combination of L-tryptophan treatment and a high fat and high fructose diet exacerbated the hepatic steatosis [29]. Therefore, further experiments will be required to clarify the role of IDO and the L-kynurenine/L-tryptophan pathway in the development of hepatic steatosis. Furthermore, after 26 weeks of being fed the HFD, the IDO-KO mice showed lower steatosis and oxidative stress than the IDO-WT mice. The hepatocyte ballooning, which indicates hepatocyte injury, was also decreased in IDO-KO mice compared to IDO-WT mice. These findings seem paradoxical given the enhanced inflammation and fibrosis in IDO-KO mice in response to the HFD. A possible explanation might be that the liver inflammation proceeded earlier in IDO-KO mice, in a similar manner to NAFLD in the clinical setting, where many cases with NAFLD show the disappearance of steatosis during its natural history, while exhibiting severe fibrosis and cirrhosis in the late stages [30,31]. In order to verify this possibility, time course studies that evaluate the levels of hepatic injury, steatosis, and inflammation caused by HFD in the early phase should be conducted. In addition, a recent study revealed that hepatic fat deposits were broken down to provide energy for fibrogenesis in a CCl_4_-treated mouse model [32]. Such a mechanism might have also been active in our HFD-fed IDO-KO mice, but again, further experiments will be required to confirm this hypothesis.

In conclusion, we herein demonstrated that IDO deficiency worsens hepatic and WAT inflammation in mice fed an HFD. Our findings suggest that regulation of the IDO-mediated immune response might be an interesting strategy for managing steatosis-related hepatic injury.
